# Inhibition of Astrocyte Connexin 43 Channels Facilitates the Differentiation of Oligodendrocyte Precursor Cells Under Hypoxic Conditions In Vitro

**DOI:** 10.1007/s12031-018-1061-y

**Published:** 2018-04-05

**Authors:** Qiong Wang, Zhen Wang, Yeye Tian, Huaqiu Zhang, Yongkang Fang, Zhiyuan Yu, Wei Wang, Minjie Xie, Fengfei Ding

**Affiliations:** 1 Department of Neurology, Tongji Hospital, Tongji Medical College, Huazhong University of Science and Technology, Wuhan, 430030 People’s Republic of China; 2 Department of Neurosurgery, Tongji Hospital, Tongji Medical College, Huazhong University of Science and Technology, Wuhan, 430030 People’s Republic of China; 3 Key Laboratory of Neurological Diseases of Chinese Ministry of Education, the School of Basic Medicine, Tongji Medical College, Huazhong University of Science and Technology, Wuhan, 430030 People’s Republic of China

**Keywords:** Astrocyte, Oligodendrocyte precursor cells, Oligodendrocyte, Connexin43, Glutamate, Hypoxia

## Abstract

Oligodendrocyte precursor cells (OPCs) proliferation and differentiation are essential for remyelination after white matter injury. Astrocytes could promote oligodendrogenesis after white matter damage whereas the underlying mechanisms are unknown. In this study, the role of astrocytic connexin43 (Cx43) hemichannels involved in OPC proliferation and differentiation in chronic hypoxia was evaluated. In an astrocyte-OPC co-culture chronic hypoxia model, OPCs became proliferative but failed to mature into oligodendrocytes. Application of astrocytic Cx43 blockers attenuated astrocyte activation, suppressed Cx43 hemichannel uptake activity and glutamate release induced by hypoxia, as well as improved OPC differentiation. Moreover, AMPA but not NMDA glutamate receptor antagonist rescued OPC differentiation in hypoxia. In conclusion, these findings suggested that astrocytic Cx43 hemichannel inhibition could potentially improve OPC maturation by attenuating AMPAR-mediated glutamate signaling. Astrocytic Cx43 hemichannels could serve as a potential therapeutic target for remyelination after chronic hypoxia.

**Electronic supplementary material:**

The online version of this article (10.1007/s12031-018-1061-y) contains supplementary material, which is available to authorized users.

## Introduction

Oligodendrocyte precursor cells (OPCs) are immature forms of oligodendrocytes (OLs) and play essential roles in remyelination after white matter injury. Remyelination involves proliferation, migration, and differentiation of OPCs, which is tightly regulated by a complex of intrinsic and extrinsic factors (Rowitch and Kriegstein 2010), including a number of neurotransmitters (De Angelis et al. 2012; Gudz et al. 2006; Wake et al. 2011; Zonouzi et al. 2011) and neurotrophins (Girard et al. 2005; McTigue et al. 1998; Murtie et al. 2005; Woodruff et al. 2004).

Astrocytes have been believed to be important in oligodendrogenesis following white matter damage (Miyamoto et al. 2015). In mammalian, astrocytes couple to neighboring cells via gap junction channels (connexin 43, connexin 30, etc), forming extensive intercellular networks. Particularly, astrocytes connect oligodendrocytes by forming gap junction. Cx43 is predominant in astrocytes, and it can form hemichannels which can release ATP, glutamate, and other small molecules under ischemic conditions (Butt et al. 2014; Li et al. 2015; Rouach et al. 2002; Schulz et al. 2015). Astrocytic Cx43 has been shown required for OPC proliferation and differentiation, which is critical for myelin maintenance (Niu et al. 2016; Orthmann-Murphy et al. 2008; Tress et al. 2012). Deletion of astrocytic Cx43 inhibited OPC proliferation (Niu et al. 2016) while Cx43/Cx32 double-knockout mice developed white matter damage at early age (Tress et al. 2012). Our previous study showed that knockout of astrocytic Cx43 significantly attenuated neuronal loss in MCAO models (Xie et al. 2011). However, it has not been studied if inhibiting astrocytic Cx43 is also protective for myelin maintanence and remyelination after ischemic injury. In the current study, we test if astrocytic Cx43 hemichannels are involved in OPC proliferation and differentiation during chronic hypoxia in astrocyte-OPC co-culture model.

## Materials and Methods

### Astrocyte and Oligodendroglia Cell Cultures

Primary astrocyte cultures were prepared as previously described (Rovegno et al. 2015; Wu et al. 2013; Yi et al. 2017) with minor modifications. Briefly, P2 newborn C57BL/6 mice cortices were dissected with the scissors. After removing meninges, the cortices were mechanically dissociated into small fragments and digested with 0.25% trypsin (Invitrogen, USA) for 5 min at 37 °C. Then, the cells were centrifuged and suspended in Dulbecco’s Modified Eagle’s Medium and Ham’s F-12 (DMEM/F12, Hyclone, USA). Next, the isolated cells were seeded at a density of 1 × 10^6^ per 25-cm^2^ flask which was coated with poly-D-lysine (Sigma-Aldrich, USA) and added DMEM/F12 supplemented with 10% fetal bovine serum (FBS, Hyclone, USA). These cultures were kept at 37 °C in an incubator (Thermo Fisher Scientific, USA) containing 95% air and 5% CO2. Purified astrocytes were obtained from primary sub-confluent cultures after removing microglia cells by shaking at 220 rpm and pre-plating for 30 min. Purified astrocytes were seeded at 3 × 10^4^/cm^2^ onto poly-D-lysine-coated glass coverslips and at 3 × 10^5^ into six-well plates. All media were changed every 2 days until the next experiment was performed. In purified cultures, more than 96% cells were glial fibrillary acidic protein (GFAP)-positive and IBA-1-positive cells were less than 3%.

The OPC cultures were prepared as previously described (Miyamoto et al. 2013; Niu et al. 2012) with minor modifications. Briefly, the mixed glial cells were isolated from P2 newborn C57BL/6 mice cerebrum and cultured with DMEM/high glucose (Hyclone, USA) supplemented with 20% FBS. Half of the culture medium was changed every other day. When the cultures were enriched with OPC cells (at 10–12 days), mixed cells were shaken (220 rpm, 2 h) to remove microglia and then shaken at 250 rpm at 37 °C for 18–20 h to harvest purified OPCs. Up to 97% harvested OPCs were positive platelet-derived growth factor receptor-α (PDGFR-α). Purified OPCs were plated on top of astrocytes at 3 × 10^4^ cells per well to construct an astrocyte-OPC co-culture system. To differentiate OPCs into mature OLs, purified OPCs or co-culture system were incubated with DMEM/F12 containing N2 (1%; Gibco, USA), B27 (1%; Gibco, USA), CNTF (10 ng ml^−1^; Peprotech, USA), T3 (50 ng ml^−1^; Sigma-Aldrich, USA), and 5% FBS, for 7 days.

### Chronic Hypoxia Model

To mimic mild chronic hypoxic conditions, astrocyte-OPC co-culture cells were incubated with non-lethal CoCl_2_ (5 μM; Sigma-Aldrich, USA) as described previously (Miyamoto et al. 2013, 2015) during OPC differentiation period. Chronic hypoxic conditions were confirmed by enhanced expression of hypoxia-inducible factor 1-alpha (HIF-1α), which is an identified marker indicating hypoxic injury (Miyamoto et al. 2015).

### Immunofluorescence Staining

Cells were washed with 0.01 M phosphate-buffered saline (PBS) 3 times and fixed with 4% paraformaldehyde for 15 min. Nonspecific binding was blocked with 10% bovine serum albumin for 1 h at room temperature (RT). Cells were then incubated overnight at 4 °C with primary antibodies diluted in PBS containing 5% bovine serum albumin. The primary antibodies include anti-glial fibrillary acidic protein (anti-GFAP; 1: 200; 3670, Cell Signaling technology, USA), anti-connexin-43 (anti-Cx43; 1: 200; AB1727, Millipore, USA), anti-myelin basic protein (anti-MBP; 1: 200; MAB386, Millipore, USA), anti-oligodendrocyte transcription factor 2 (anti-Olig2; 1: 200; AB9610, Millipore, USA), anti-HIF-1α (1: 100; ab113642, Abcam, USA), and anti-neuroglial antigen 2 (anti-NG2; 1: 200; AB5320, Millipore, USA). For negative controls, the same source as primary antibodies nonspecific IgG was used instead of the primary antibody, and positive controls were performed and compared as those reported previously. After washing three times, the cells were incubated for 1 h at RT with either cyanine3-conjugated goat anti-rabbit IgG (1: 400; 111-165-144, Jackson-ImmunoResearch, USA), fluorescein isothiocyanate-conjugated goat anti-mouse IgG (1: 400; 115-165-146, Jackson-ImmunoResearch, USA), or cyanine3-conjugated goat anti-rat IgG (1: 400; 112-165-143, Jackson-ImmunoResearch, USA). The nuclei were stained with 4,6-diamidino-2-phenylindole (DAPI; 1: 1000; D9542, Sigma, USA). The images were captured using a fluorescent microscope (BX51; Olympus, Japan) and a laser confocal microscope (LSM1200; Olympus, Japan). Cells were counted in visual field by Image J.

### Western Blotting

Cells were collected in radioimmunoprecipitation assay (RIPA) buffer supplemented with protease inhibitor cocktail (Roche, USA), and nucleoproteins were extracted through nuclear extraction kit (p0028, Beyotime, China). Equal amounts of proteins (20–30 μg) of each group were resolved on a 10–15% SDS-PAGE gel and subsequently transferred to a polyvinylidene difluoride membrane (0.22 μm; Millipore, USA) using a semidry Trans-Blot system (Bio-Rad laboratories, USA). The membranes were blocked for 1 h at RT in Tris-buffered saline containing 0.25% Tween-20 (TBST) and 5% non-fat milk. The membranes were then incubated with primary antibodies overnight at 4 °C. Primary antibodies included anti-GFAP (1: 1000, ab7260, Abcam, USA), anti-Cx43 (1: 500), anti-MBP (1: 500), anti-PDGFR-α (1: 1000; 558774, BD Biosciences, USA), anti-HIF-1α (1: 1000), anti-Histone H3 (1:10000; GB13102-1, servicebio, China), anti-Glyceraldehyde 3-phosphate dehydrogenase (GAPDH; 1: 1000; sc-66163, Stanta Cruz, USA), and anti-β-Actin (1: 1000; sc7210, Stanta Cruz, USA). After washing four times with TBST, the membranes were incubated with Odyssey secondary antibody-IRDye 800-conjugated anti-rabbit IgG or Alexa Fluor 700-conjugated anti-mouse IgG (1: 10000; 925-32211, 926-68070, LI-COR Bioscience, USA) for 1 h at RT. The immunoreactivity of protein bands was captured using an Odyssey IR imaging system (LI-COR Bioscience, USA). The images were analyzed with Image J to obtain the integrated optical density (OD) of signals. GAPDH, β-Actin, and Histone H3 were used as loading controls.

### EtBr Uptake Analysis

To assess Cx43 hemichannel activity, cells were incubated with ethidium bromide (EtBr; Sigma-Aldrich, USA) as described (Giaume et al. 2012; Yi et al. 2017) with minor modifications. In brief, co-cultured cells were incubated with 5 μM EtBr for 10 min at 37 °C under different treatment conditions. The cells were then washed three times with PBS and immediately fixed with 4% paraformaldehyde for 15 min at RT. Immunofluorescence was captured using a confocal laser-scanning microscope at an excitation wavelength of 555 nm. The amount of EtBr uptake was evaluated by calculating fluorescence-positive area per visual field with Image J.

### 5-Ethynyl-20-Deoxyuridine Incorporation Assay

The 5-ethynyl-20-deoxyuridine (EdU) assay kit (Ribobio, China) was used to examine proliferation of OPCs according to manufacturer’s instructions. After being incubated with the proliferation media for 3 days, co-cultures were treated with differentiation media supplemented with EdU (50 μM) for another 24 h at 37 °C, and then washed three times with PBS and fixed with 4% paraformaldehyde for 15 min at RT. Cells with EdU were labeled as instructions. After the cells were incubated with the primary Olig2 (1: 200) and the secondary antibody (fluorescein isothiocyanate-conjugated goat anti-rabbit IgG), images were captured with a confocal laser-scanning microscope. Subsequent image analysis with Image J included cell counts. The OPC proliferation ratio was calculated as the percentage of the number of EdU and Olig2 double-positive cells out of Olig2-positive cells.

### Glutamate Concentration Analysis

Glutamate levels were tested with glutamic acid kit (Nanjing Jiancheng Bioengineering Institute, China) in collected culture media of different time points during OPC differentiation stage. The sample OD values were detected with a BioSpectrometer (Eppendorf, Germany) and used to calculate the final concentration of glutamate (μmol L^−1^).

### Statistical Analysis

All the data are presented as the mean ± standard deviation (SD) from at least three independent experiments. Data were analyzed with GraphPad Prism 7 software (GraphPad Software, USA). Statistical comparisons were made using Student’s *t* test or ANOVA with Tukey post hoc analysis for multiple comparisons. A *p* value < 0.05 was considered statistically significant.

## Results

### Astrocyte-OPC Co-Cultures Under Chronic Hypoxic Conditions In Vitro

**Fig. 1:**
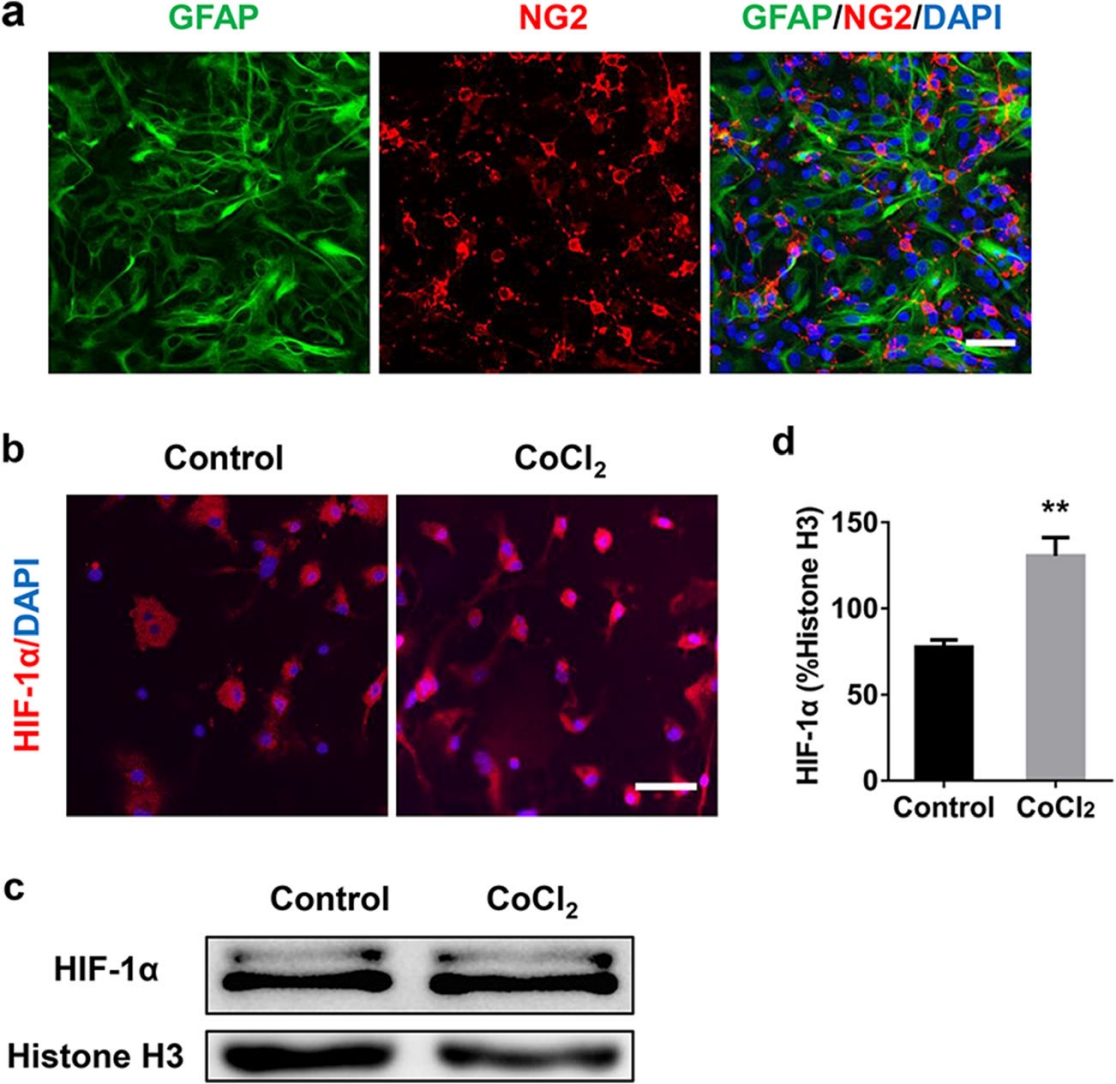
Astrocyte and OPC co-culture system of chronic hypoxia model. **a** Representative images of GFAP (astrocyte marker; green) and NG2 (OPCs marker; red) in the co-culture model. The cell nuclei were stained with DAPI (blue). **b** Sublethal CoCl_2_ (5 μM) was administered to mimic prolonged hypoxia in vitro and resulted in the translocation of the hypoxic marker HIF-1α from cytoplasm into to the nuclei in co-cultures. **c**, **d** Western blot analysis demonstrated an increased expression of HIF-1α by nucleoprotein analysis, histone H3 was used as a loading control. Scale bar, 50 μm. Data are mean ± SD, **p < 0.01, control group vs CoCl_2_ group by Student’s *t* test

We developed an astrocyte-OPC co-culture model and validated the model by staining specific markers for astrocytes (GFAP, green) and OPCs (NG2, red) (Fig. 1a). In order to mimic chronic hypoxia, co-culture cells were exposed for 7 days to a sublethal dose of CoCl_2_ (5 μM) in the differentiation media (Miyamoto et al. 2015). Compared to the control, CoCl_2_ treatment instigated HIF-1α translocation from cytoplasm to nuclei (Fig. 1b) and enhanced HIF-1α expression in nuclei (Fig. 1c, d) in co-cultures, without influencing cells viability (Fig. S1) and inducing cells death (Fig. S2).

### Cx43 Inhibitors Attenuated Hypoxia-Induced Astrocyte Activation

**Fig. 2:**
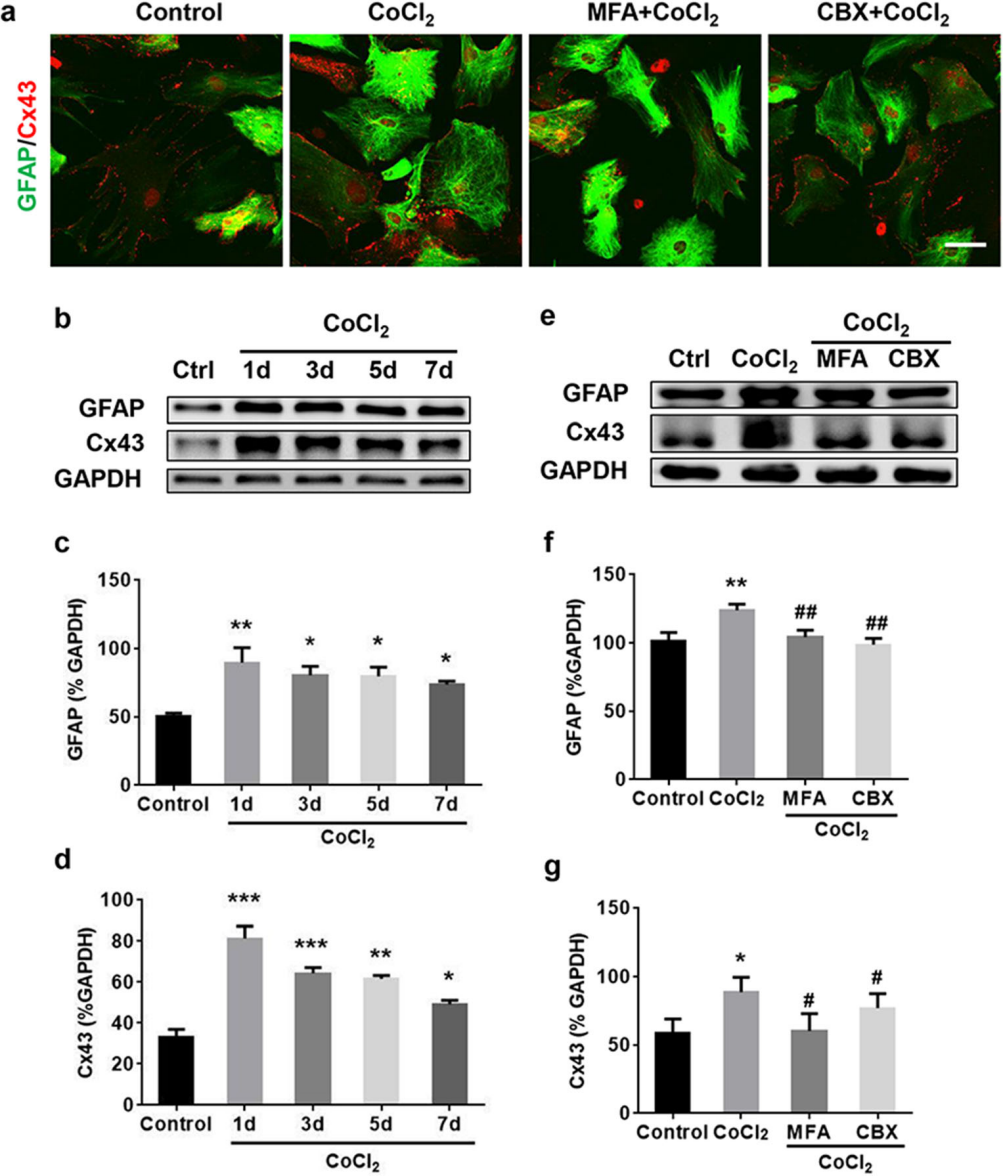
Cx43 inhibitors attenuated astrocyte activation under chronic hypoxia. **a** Representative images of activated astrocytes, with up-regulated GFAP (green) and Cx43 (red), after 2 days of hypoxia as compared to control. MFA (10 μM) and CBX (50 μM) attenuated astrocyte activation. **b**–**d** Western blotting confirmed increased GFAP and Cx43 protein levels following hypoxia. **e**–**g** MFA and CBX inhibitors decreased hypoxic-induced GFAP and Cx43 protein upregulation in CoCl_2_-treated cultures. GAPDH was used as a loading control. Scale bar, 50 μm. Data are mean ± SD; *p < 0.05, **p < 0.01, ***p < 0.001, CoCl_2_ group vs control group by one-way ANOVA; #*p* < 0.05, ##*p* < 0.01, MFA or CBX group vs CoCl_2_ group by one-way ANOVA

By double immunofluorescent staining, it revealed that Cx43 was co-localized in GFAP-positive astrocytes in co-culture, mainly in cell membrane (Fig. 2a). Compared with normoxia condition, the expression of GFAP and Cx43 was markedly upregulated by 1 day of hypoxia, and gradually but not entirely recovered over the subsequent days (3, 5, and 7 days) (Fig. 2b–d). Gap junction inhibitors meclofenamic acid (MFA, 10 μM) or carbenoxolone (CBX, 50 μM) (Fig. 2a, e–g) could significantly attenuate hypoxia-induced enhancement of GFAP and Cx43 expression at day 2 post-hypoxia treatment, without affecting cells viability (Fig. S1).

### Cx43 Inhibitors Rescued the Limited OPC Maturation Under Chronic Hypoxia

**Fig. 3:**
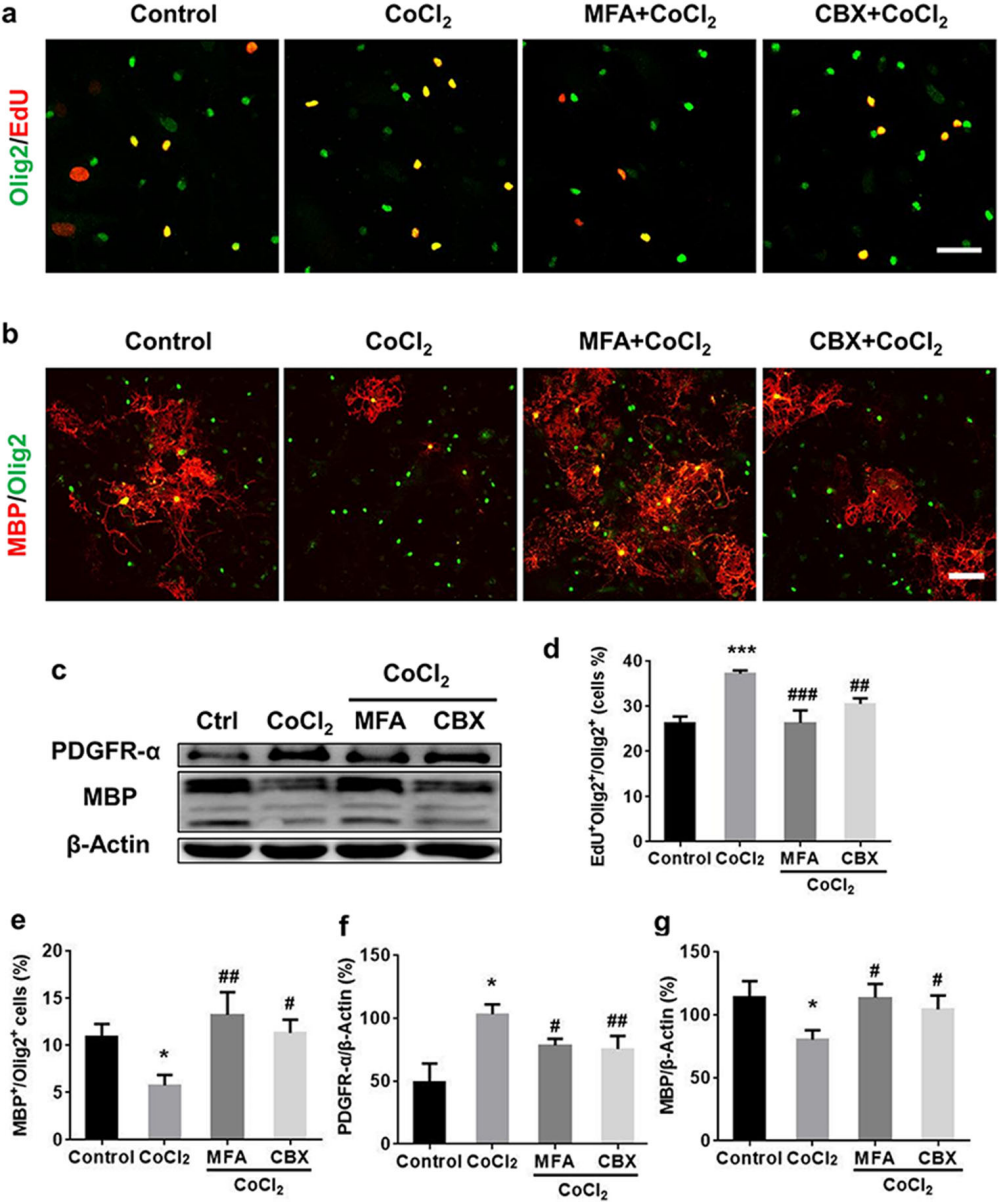
OPC maturation was suppressed under hypoxic conditions, while Cx43 inhibitors rescued OPC differentiation. **a**, **d** After 1 day of hypoxia, OPC proliferation is increased compared to control according to the percentage of EdU^+^Olig2^+^ (EdU (red), Olig2 (green)). **b**, **e** Based on MBP (red, OL) and Olig2 staining (green, oligodendroglia lineage cells), the proportion of maturing oligodendrocyte decreased after 7 days of hypoxia compared to the control. In addition, Cx43 inhibitors decreased the proliferation of OPCs (**a**, **d**) but promoted OPCs differentiation (**b**, **e**). This was confirmed by Western blotting with PDGFR-α (OPCs marker) and MBP (maturing OL marker); β-actin acted as a loading control (**c**, **f**, **g**). Scale bars, 50 μm (**a**); 100 μm (**b**). Data are mean ± SD, *p < 0.05, ***p < 0.001, CoCl_2_ group vs control group by one-way ANOVA; #*p* < 0.05, ##*p* < 0.01, ###*p* < 0.001, MFA or CBX group vs CoCl_2_ group by one-way ANOVA

The proliferating OPCs were labeled by double-staining EdU and the oligodendroglia lineage marker Olig2. A significant increase in the percentage of EdU^+^Olig2^+^ out of Olig2^+^ cells was observed after hypoxia as compared to normoxic control (Fig. 3a, d), which was inhibited by MFA (10 μM) or CBX (50 μM) treatment (Fig. 3a, d). Meanwhile, the enhanced OPC proliferation was accompanied by failure of the maturation of OLs after hypoxia, which was indicated as a remark decrease in the percentage of MBP^+^ out of Olig2^+^ cells (Fig. 3b, e). MFA and CBX treatment could rescue the reduction of MBP^+^/Olig2^+^ cell ratio (Fig. 3b, e).

In agreement with immunofluorescent staining, Western blot confirmed the hypoxia-induced upregulation of PDGFR-α (OPCs marker) was inhibited by MFA or CBX treatment (Fig. 3c, f). Meanwhile, the hypoxia-induced MBP (mature OL marker) reduction could also be rescued by MFA and CBX treatment (Fig. 3c, g). Taken together, the results indicated that Cx43 inhibitors could suppress proliferation but promote differentiation of OPCs under mild chronic hypoxia.

### Cx43 Inhibitors Suppressed Cx43 Hemichannel Activity and Glutamate Release Under Chronic Hypoxia

**Fig. 4:**
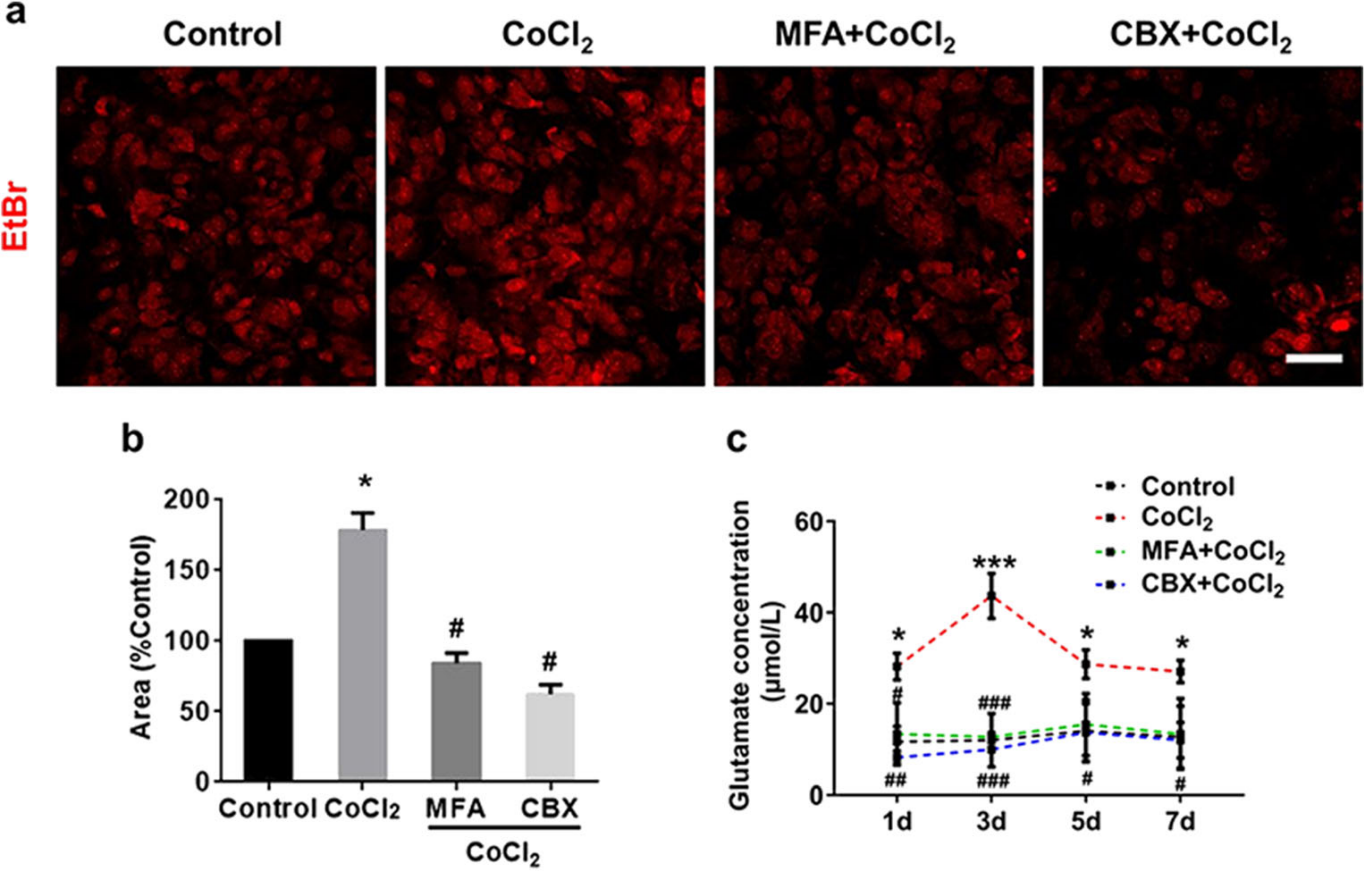
Cx43 hemichannel activity was upregulated under hypoxia. **a**, **b** Hypoxia could increase EtBr uptake in co-cultures, while MFA and CBX attenuated this effect. **c** Extracellular glutamate levels were increased during hypoxia, while MFA and CBX could markedly suppress this effect. Scale bar, 50 μm. Data are mean ± SD, *p < 0.05, ***p < 0.001, CoCl_2_ group vs control group by one-way ANOVA, #*p* < 0.05, ##*p* < 0.01, ###*p* < 0.001, MFA or CBX group vs CoCl_2_ group by one-way ANOVA

To establish the effect of Cx43 inhibitors on hemichannel activity, EtBr dye uptake was measured as previously reported (Giaume et al. 2012). Astrocyte showed that a significant increased dye uptake in hypoxia condition compared to normoxia (Fig. 4a, b). However, when incubated with Cx43 inhibitors, the hypoxia-induced dye uptake increase was significantly reduced (Fig. 4a, b). The Cx43 hemichannels are well-known to be a pathway to release glutamate (Ye et al. 2003). We found that, after 3 days of hypoxia treatment, the extracellular glutamate level was significantly higher than that under normoxic conditions (43.63 ± 4.87 versus 9.92 ± 5.84 μmol L^−1^, *p* < 0.001; Fig. 4c). At days 5 and 7 of hypoxia, the extracellular glutamate levels were relatively lower than earlier time point but remained higher than that under normoxia (28.65 ± 3.15 versus 14.00 ± 6.81 μmol L^−1^ at day 5; 27.09 ± 2.47 versus 13.30 ± 5.61 μmol L^−1^ at day 7; *p* < 0.05; Fig. 4c). MFA or CBX treatment significantly attenuated the glutamate release under chronic hypoxia at all time point (*p* < 0.05; Fig. 4c).

### AMPA Receptor Inhibitor Partially Rescued the Limited OPC Maturation Under Hypoxia

**Fig. 5:**
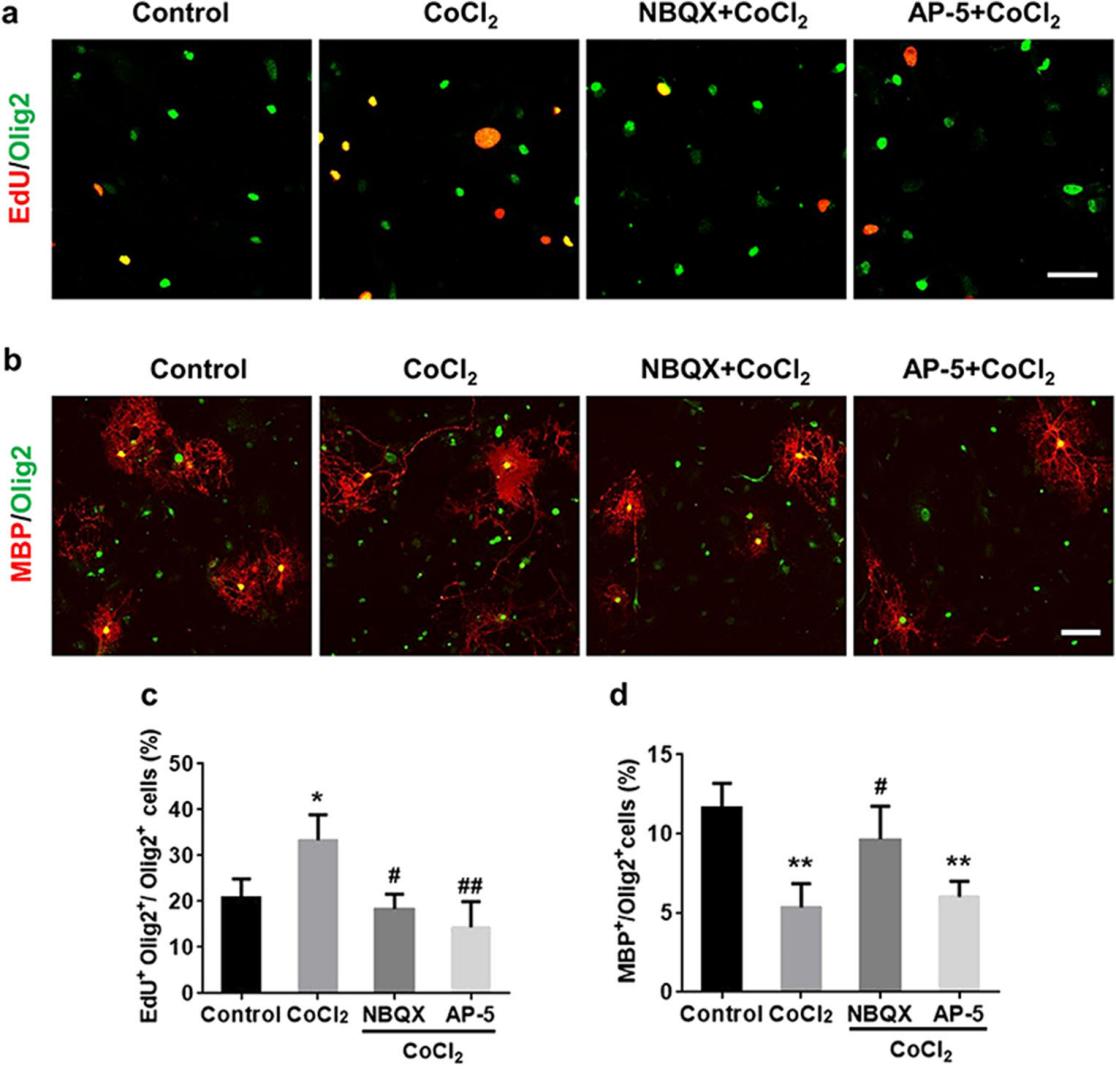
AMPAR antagonist promotes OPC maturation under hypoxic conditions. When cultures were treated with AMPAR and NMDAR antagonists (NBQX and AP-5, respectively) during hypoxia, both NBQX (10 μM) and AP-5 (50 μM) attenuated OPC proliferation (**a**, **c**). Only NBQX but not AP-5 partially rescue the limited differentiation of OPCs under hypoxia (**b**, **d**). Scale bar, 50 μm (**a**); 100 μm (**b**). Data are mean ± SD, *p < 0.05, **p < 0.01, CoCl_2_ or AP-5 group vs control group by one-way ANOVA; #*p* < 0.05, ##*p* < 0.01, NBQX or AP-5 group vs CoCl_2_ group by one-way ANOVA

Glutamate receptors are expressed in oligodendroglia lineage cells (De Biase et al. 2010; Micu et al. 2006) and have been shown to mediate excitotoxicity, migration, proliferation, and maturation of oligodendroglia lineage cells (Deng et al. 2003, 2006; Dohare et al. 2016; Li et al. 2013; Micu et al. 2006). We applied α-amino-3-hydroxy-5-methyl-4-isoxazolepropionic acid (AMPA) or N-methyl-D-aspartate (NMDA) glutamate receptor inhibitors to test if OPCs were responding to the extracellular glutamate released from Cx43 hemichannels under chronic hypoxia. AMPA receptor antagonist NBQX (10 μM) or NMDA receptor antagonist DL-AP-5 (50 μM) could significantly decrease the percentage of EdU^+^Olig2^+^/Olig2^+^ proliferating OPCs (Fig. 5a, c). Interestingly, under hypoxia, NBQX but not DL-AP-5 treatment partially improved the percentage of MBP^+^/Olig2^+^ cells (Fig. 5b, d). These results indicated that AMPAR activation suppressed OPC maturation under hypoxia.

## Discussion

In present study, we showed that inhibition of astrocytic Cx43 could suppress glutamate release via hemichannels and promote OPC maturation after chronic hypoxia injury via AMPA receptor inhibition. In an astrocyte-OPC co-culture system, hypoxia could upregulate astrocytic Cx43 expression and induce astrocyte activation, as well as glutamate release via Cx43 hemichannels. Pharmacologically inhibiting astrocytic Cx43 could significantly suppress glutamate release and therefore improve differentiation of OPCs. Furthermore, AMPA receptor but not NMDA receptor antagonist partially enhanced the maturation of OPCs under hypoxia. This study has provided new insights into the role of astrocytic Cx43 involved in OPC differentiation.

OPC proliferation and differentiation after demyelination are mediated by multiple factors, including intercellular communication (Shindo et al. 2016) and extracellular environment (Rosenzweig and Carmichael 2015). Particularly, it has been known that astrocytes regulate oligodendrogenesis by secreting trophic factors into extracellular space, such as erythropoietin under hypoxic and reoxygenation injury in vitro (Kato et al. 2011) or brain-derived neurotrophic factor (BDNF) in the cuprizone-induced demyelination model (Fulmer et al. 2014). In addition, direct astrocyte and oligodendrocyte communication via gap junctions are essential for the proper functioning of myelination (Orthmann-Murphy et al. 2008). Furthermore, our group has found that inhibition of Cx43 channels could be protected for myelin sheath damage in chronic cerebral hypoperfusion mice model (unpublished data). Our study showed that astrocytic Cx43 hemichannels could release glutamate which resulted in AMPA receptor-mediated inhibition of OPC maturation after prolonged hypoxia in vitro.

Gap junctions are involved in maintaining myelin homeostasis. Astrocyte primarily expresses Cx43 and Cx30, whereas oligodendrocytes express Cx47, Cx32, and Cx29 (Orthmann-Murphy et al. 2008). Double knockout of Cx32 and Cx43 could give rise to white matter vacuolation and progressive loss of astrocytes and induce sensorimotor impairment, seizure activity, and early mortality (Magnotti et al. 2011). Mice lacking both astrocytic Cx30 and 43 exhibited myelin degeneration and vacuole formation while deletion either of these connexins could retain myelin integrity (Cotrina and Nedergaard 2012; Lutz et al. 2009). Deletion of astrocytic Cx43 affected OPC proliferation due to altered glucose support from astrocytes (Niu et al. 2016). Furthermore, peptide5, which blocks the Cx43 hemichannels, was shown to improve oligodendrocyte survival after brain ischemia (Davidson et al. 2012, 2013). Our results indicated that the inhibition of astrocytic Cx43 could be beneficial for facilitating OPC maturation after chronic hypoxia.

Cx43 hemichannels act as important pathway for release of glutamate, ATP, cytokines, playing an active role in neuroinflammation (Rouach et al. 2008). Accumulating evidence indicates a key role for glutamate signaling in white matter pathology (Butt et al. 2014). Glutamate-evoked Ca^2+^ signals in oligodendrocytes are closely related with OL differentiation and myelination, although the expression of AMPARs and NMDARs on oligodendroglia lineage cells is still undefined (De Biase et al. 2010; Li et al. 2013). It is thought that AMPARs were mainly expressed on OPCs and were downregulated in immature OL and OL. NMDARs, on the other hand, are believed to be restricted to immature OL and OL with generally low expressions, but absent in OPCs. White matter OPCs expressing AMPARs can be activated by glutamate (Hamilton et al. 2010). Glutamate released from axons could promote myelination of OL lineage cells during postnatal development (Kougioumtzidou et al. 2017). However, AMPAR activation in oligodendrocytes has also been shown to cause cell death and inhibit OPC maturation as well as remyelination in hypoxia-ischemia models (Follett et al. 2004; Volpe 2009). Meanwhile, it has been reported that NMDAR signaling was not required for OPC development (De Biase et al. 2011). Our data suggested that administration of an AMPAR but not NMDAR antagonist reduced OPC proliferation and promoted their differentiation into OLs.

**Fig. 6:**
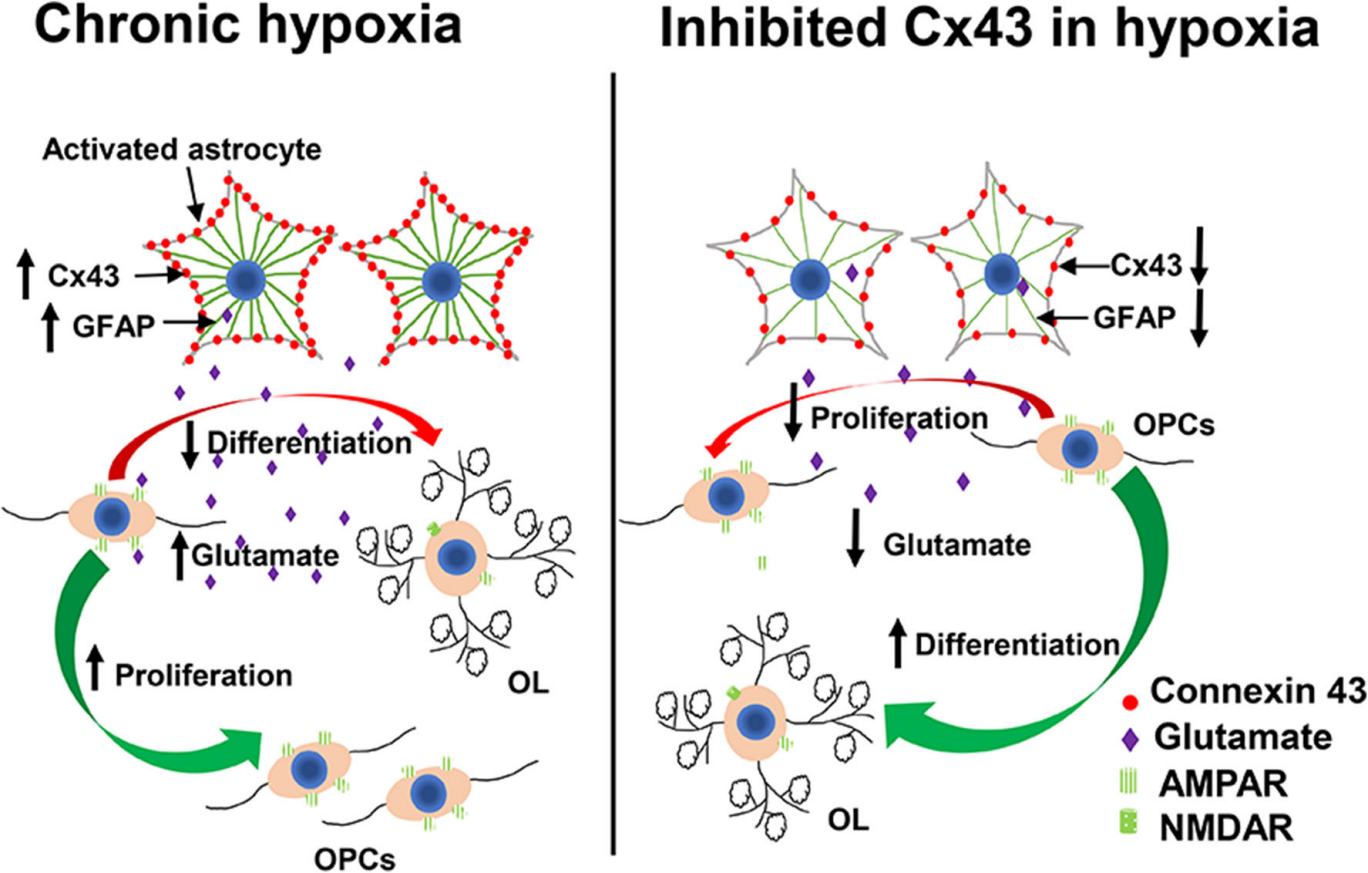
Schematic model that astrocytic Cx43 hemichannels facilitate OPC differentiation under chronic hypoxia via AMPAR-mediated glutamate signaling

In summary, astrocytic Cx43 hemichannels could result in increase in glutamate release after chronic hypoxia, which could impair OPC maturation and remyelination processes. Glutamate released via Cx43 hemichannels suppressed OPC differentiation partially mediated by AMPAR activation (Fig. 6). Application Cx43 inhibitors could rescue hypoxia-induced suppression of OPC differentiation. Based on our findings, astrocytic Cx43 hemichannels could potentially be a therapeutic target for facilitating OPC remyelination in hypoxia-induced white matter injury.

## Electronic Supplementary Material

ESM 1 (DOC 365 kb)
