# Supplementary material for: Inhibition of Astrocyte Connexin 43 Channels Facilitates the Differentiation of Oligodendrocyte Precursor Cells Under Hypoxic Conditions In Vitro
Source: J Mol Neurosci. 2018 Apr 5;64(4):591–600. doi: 10.1007/s12031-018-1061-y (PMC6763517; doi:10.1007/s12031-018-1061-y)
Supplement: Supplementary file 1 — (DOC 365 kb) [file 12031_2018_1061_MOESM1_ESM.doc]

**Supplementary material**

**CCK-8 assay**

CCK-8 kit (Beyotime, China) was used to assessment of cells viability of different drugs administration to choose appropriate concentration following-up experiment. Briefly, both astrocytes and OPCs cells were planted at 3000 cells/100μl into 96-well cultured plate with completed medium for 48h. Then, cells were exposed to various intervention condition continuing incubation 24h. Later, CCK-8 reagent was added to each well and incubation for 0.5-3h before measurement of optical density (OD) with a microplate reader at the excitation wave of 450nm, reference wave of 600nm. Experiment repeated three times. Cells viability was expressed as OD values.

**Lactate dehydrogenase (LDH) release assay**

Cells injury were quantitatively assessed by measuring LDH released from damaged or dead cells in co-culture system using the LDH assay kit (Beyotime, China) following the manufacturer’s instructions. Briefly, both astrocytes and oligodendrocytes were seeded at 3000 cells/100ul in 96-well plates and incubated for 48 h. Then, cells were exposed to various intervention condition continuing incubation 24h. After that, 120 µL medium was collected and the amount of LDH leakage from the cells was determined at 490 nm using a microplate reader (TECAN). The results were expressed as OD values.

**Terminal deoxynucleotidyl transferase-mediated dUTP nick end-labeling (TUNEL) staining**

An apoptosis detection kit (Clontech, USA) was used for TUNEL staining according to the manufacturer’s instruction. Briefly, co-cultures were fixed by 4% PFA/PBS, then incubated with 0.25% Triton X-100 for 5min at RT, followed with equilibration buffer for 10 min at RT, and treated with TdT incubation buffer for 60 min at 37°C. Cells were also stained with DAPI to label nuclear in situ. Images were captured by confocal laser-scanning microscope, and counted with Image J software. Quantification of TUNEL staining on each group in terms of percentage of TUNEL-positive cells number relative to total cells number.


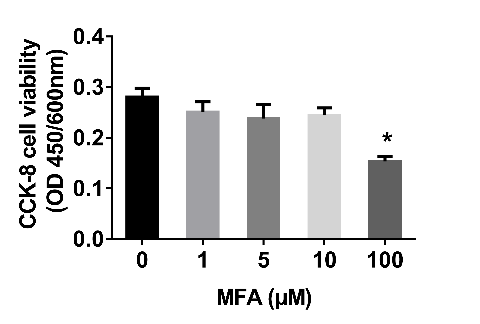


**b**


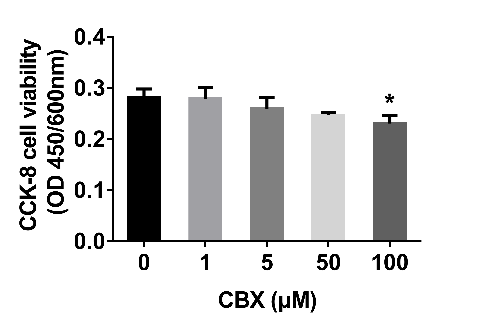


**c**


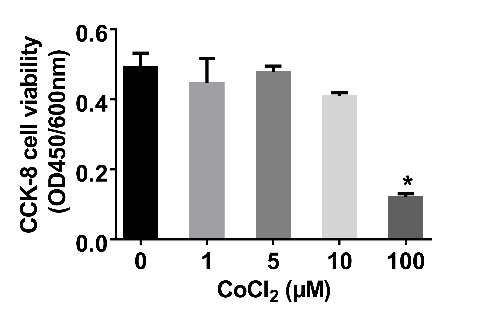


**a**

**d**


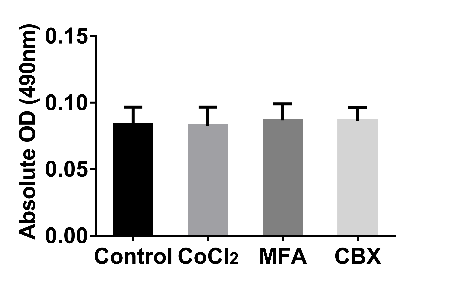


Supplementary figure1 Cells viability were detected in astrocytes and OPCs co-cultures co-culture system under different treatments. (**a-c**) CCK-8 assay kit was used to assessment the cell viability under different treatments in different concentrations to choose optimal concentration for following study. Cells were administrated in each condition with 48h before measurement. Then 5μM CoCl_2_ (**a**), 10μM MFA (**b**) and 50μM CBX (**c**) were chosen as concentrations of intervention. (**d**) Lactate dehydrogenase (LDH) release rates measured using an LDH kit showed that CoCl_2_ (5μM), MFA (10μM) and CBX (50μM) had no effect on cells viability. Data are mean ± SD, *p <0.05, CoCl_2_ or MFA or CBX group vs control group by one-way ANOVA.


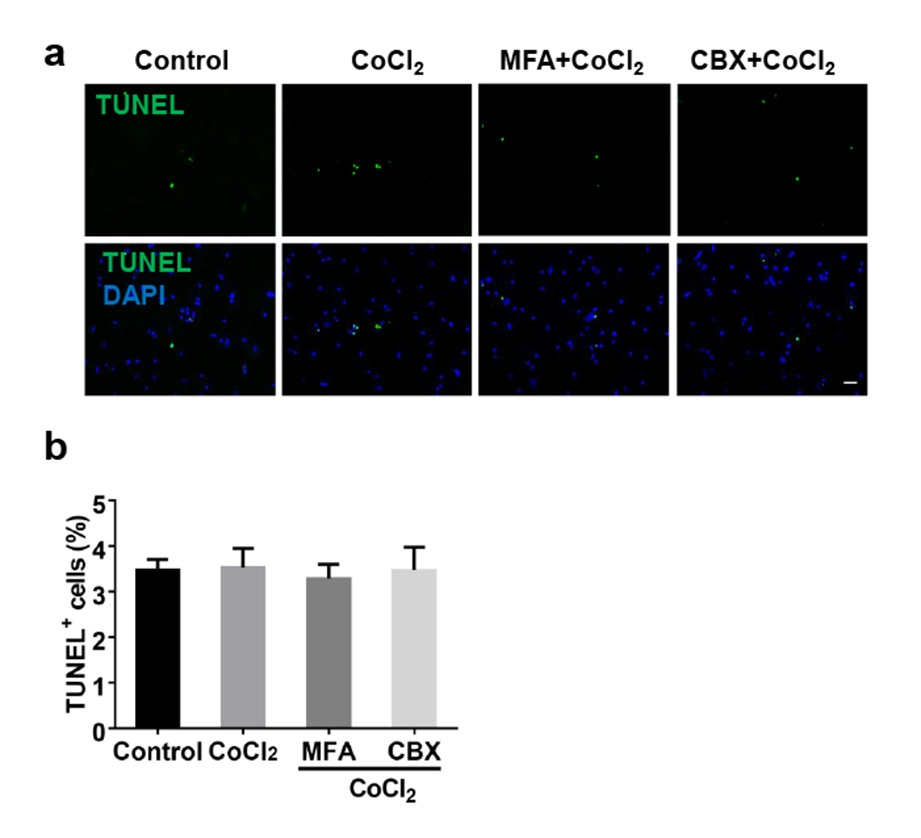


Supplementary figure2 Various treatments with CoCl_2_ (5μM) and MFA (10μM) or CBX (50μM) did not cause significant changes in cells apoptosis compared with that in control detected by TUNEL staining. (**a-b**) Administrated. Scale bar, 50μm. Data are mean ± SD, CoCl_2_ or MFA or CBX group vs control group by one-way ANOVA.
